# Management of complicated diverticulitis of the colon

**DOI:** 10.1002/ags3.12035

**Published:** 2017-09-28

**Authors:** Toru Tochigi, Chihiro Kosugi, Kiyohiko Shuto, Mikito Mori, Atsushi Hirano, Keiji Koda

**Affiliations:** ^1^ Department of Surgery Teikyo University Chiba Medical Center Ichihara Chiba Japan

**Keywords:** colonic diverticulitis, complicated diverticulitis of the colon, Hinchey classification

## Abstract

Diverticular disease of the colon occurs quite frequently in developed countries, and its prevalence has recently increased in Japan. The appearance of diverticulosis increases with age, although mostly remaining asymptomatic. Approximately 20% of cases require treatment. As the Western lifestyle and number of elderly people increase, the need for medical treatment also increases. Computed tomography (CT) is the gold standard for diagnosing diverticulitis. Complicated diverticulitis is classified by the size and range of abscess formation and the severity of the peritonitis. Each case should be classified based on clinical and computed tomography (CT) findings and then treated appropriately. Most patients with uncomplicated diverticulitis (stages 0–Ia) can be treated conservatively. Diverticulitis with a localized abscess (stages Ib–II) is generally resolved with conservative treatment. If the abscess is larger or conservative treatment fails, however, percutaneous drainage or surgery should be considered. Operative treatment is considered standard therapy for severe diverticulitis with perforation and generalized peritonitis (stages III–IV). Colonic diverticulitis treated conservatively frequently recurs. Elective surgery after recovery should be considered carefully and decisions made on a case‐by‐case basis. Because cases of colonic diverticulitis will undoubtedly increase in Japan, it is likely that we will be confronted with increasing numbers of treatment decisions. We therefore need to have a systematic strategy for treating the various stages of colonic diverticulitis appropriately. We herein review the management of complicated diverticulitis.

## 1 INTRODUCTION

Colonic diverticula are small outpouchings from the colonic lumen owing to mucosal herniation through the colonic wall at sites of vascular perforation.^1^ Colonic diverticulosis occurs quite frequently in developed countries, and an increasing prevalence of the disease has been reported in Japan. Whereas its prevalence in Japan in 1980 was reported to be only 5.5%,^2^ during the 1990s it was variously reported at 10.9‐39.7%.^3^ The prevalence of diverticulosis also increases with age, from 16‐22% in people <40 years of age to 42‐60% in those >80 years.^4^ The increased appearance of diverticulosis in Japan has been attributed to an increasingly westernized lifestyle^5^ and the advanced age of the overall population.

There is a difference in the localization of diverticula between Western and Asian countries. Colonic diverticula are found in the sigmoid colon in >90% of patients in Western countries, whereas the right‐sided type is found in about 70% of the patients in Japan.^6^ A multi‐institutional study in Japan revealed that the right‐sided type accounted for 69.3%, the bilateral type for 16.8%, and the left‐sided type for 13.9%. There is a high frequency of the right‐sided type during middle age, whereas the left‐sided and bilateral types increase with age, with about half occurring in people >70 years.^7^

Approximately 75‐80% of patients with anatomical diverticulosis remain asymptomatic throughout their lifetime. For the other approximately 20% of patients with diverticulosis who develop diverticulitis,^1^ 1‐2% require hospitalization, and 0.5% require surgery because of complications such as an abscess or peritonitis.^8^ Herein, we review the management of complicated diverticulitis.

## 2 DIAGNOSIS

For patients in whom complicated diverticulitis is suspected, the precise diagnosis requires cross‐sectional imaging such as computed tomography (CT).^9^ With its reported 93‐97% sensitivity and 100% specificity, CT has evolved as the gold standard diagnostic test for suspected diverticulitis.^10^, ^11^ CT is minimally invasive, rapid, and widely available. It is not only useful for diagnosis, but also for the observation of changes over time.^12^ It provides details regarding the size and location of an abscess, providing an objective analysis of the severity of the diverticulitis. Treatment decisions can then be made based on CT scans. Ultrasonography serves as another good diagnostic tool and may supplement the information obtained from CT,^13^, ^14^ although its usefulness depends on the experience of the examiner.^15^, ^16^

## 3 CLASSIFICATION

**Figure 1:**
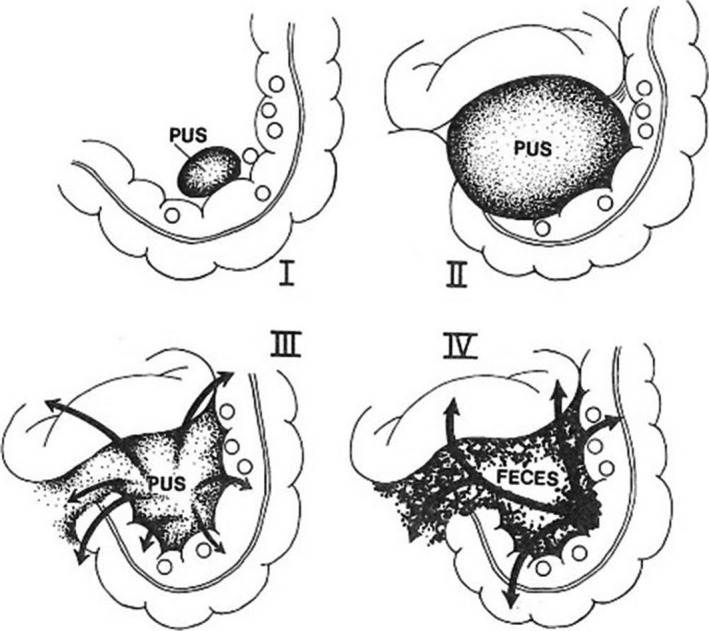
The four clinical stages of perforated diverticular disease. Stage I: This is a pericolic abscess confined by the mesentery of the colon. Stage II: This is a pelvic abscess resulting from local perforation of a pericolic abscess. Stage III: This is generalized peritonitis resulting from the rupture of either a pericolic or a pelvic abscess into the general peritoneal cavity. Stage IV: Fecal peritonitis results from the free perforation of a diverticulum. (From Hinchey et al.^17^)

The Hinchey classification (Figure 1)^17^ has traditionally been used to distinguish four stages of complicated diverticulitis. It is well known and, to date, still widely used. Originally, the Hinchey classification was intended to be used during laparotomy. As advanced imaging modalities and conservative treatments have improved, however, this classification has been considered increasingly unsuitable for determining the treatment strategy for complicated diverticulitis. Hence, modified versions of the Hinchey classification have been reported and are currently used in clinical practice.

Kaiser et al^18^ proposed a modified Hinchey classification in 2005, wherein the size and range of abscess formation and the severity of peritonitis are taken into account (Table 1). These authors introduced stage 0, defined by clinically mild diverticulitis, and differentiated stage I into stage Ia (limited pericolonic inflammation) and stage Ib (abscess formation). This broadening of the original Hinchey classification not only addresses perforated diverticulitis, but also includes mild clinical disease. Dharmarajan et al^19^ reported another CT grading system for perforated diverticulitis in 2011 that reflects the amount and location of extraluminal air and the size of the abscess. Here, the amount of free air and the size of the abscess are determined and divided by 2 and 4 cm, respectively. This system is based on a study by Siewert et al,^20^ who said that an abscess of >4 cm requires percutaneous drainage, whereas smaller abscesses can be treated with antibiotic therapy alone.

**Table 1:** Classifications of diverticulitis and its CT findings

Hinchey classification		Modified Hinchey classification		Accompanying CT findings
		Stage 0	Clinically mild diverticulitis	Diverticula with or without wall thickening of the colon
Stage I	Pericolic abscess or phlegmon	Stage Ia	Confined pericolic inflammation and phlegmonous inflammation	Colonic wall thickening with inflammatory reaction in pericolic fatty tissue
		Stage Ib	Abscess formation (<5 cm) in the proximity of the primary inflammatory process	Alterations as stage Ia + pericolic or mesocolic abscess formation
Stage II	Pelvic, intra‐abdominal, or retroperitoneal abscess	Stage II	Intra‐abdominal abscess, pelvic or retroperitoneal abscess, abscess distant from the primary inflammatory process	Alteration as stage Ia + distant abscess formation (mostly pelvic or interloop abscesses)
Stage III	Generalized purulent peritonitis	Stage III	Generalized purulent peritonitis	Free air with local or generalized free fluid and possible thickening of the peritoneum (no open communication with bowel lumen)
Stage IV	Generalized fecal peritonitis	Stage IV	Fecal peritonitis	Free perforation, open communication with bowel lumen

Computed tomography (CT) findings according to Kaiser et al.^18^

**Figure 2:**
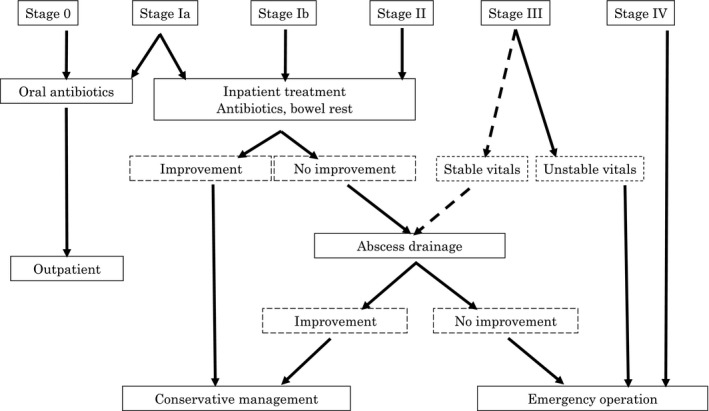
Protocol for management of complicated diverticulitis of the colon. Based on the modified Hinchey classification, diverticulitis may be categorized into six stages (0, Ia, Ib, II, III, IV). Each case should be treated appropriately and the treatment options are shown in this flowchart

## 4 TREATMENT

The medical treatment strategy of a new patient with acute diverticulitis of the colon should be based on whether the peritonitis has remained local or has spread diffusely into the abdominal cavity. In patients whose vital signs are stable and abdominal symptoms are localized, conservative treatment may be successful even if imaging reveals free air in the abdominal cavity. However, when diffuse peritonitis is suspected based on the abdominal examination, emergency surgery may be required even if imaging shows that the abscess is localized. A decision that surgery is necessary is based on the patient's vital signs, laboratory data, and abdominal symptoms. Even when conservative therapy is selected, frequent checking of its efficacy is needed so as not to lose the appropriate timing of surgery, if it becomes necessary. Based on the proposed classification, diverticulitis may be categorized into six stages (0, Ia, Ib, II, III, IV) based on the clinical and CT findings as follows. The treatment options described here are shown in Figure 2.

### 4.1 Stages 0–Ia

More than 70% of patients with acute diverticulitis display no evidence of an abscess or perforation.^18^ Diverticulitis can be diagnosed based only on clinical findings (abdominal pain, fever, leukocytosis) (stage 0), or CT may reveal phlegmonous changes (stage Ia). Most patients with uncomplicated diverticulitis (stage 0 or Ia) are treated conservatively, with a success rate of 93‐100%.^21^ Most patients can be treated on an outpatient basis. These patients respond well to antibiotics that are effective against Gram‐positive, Gram‐negative, and anaerobic bacteria.

### 4.2 Stages Ib–II

**Figure 3:**
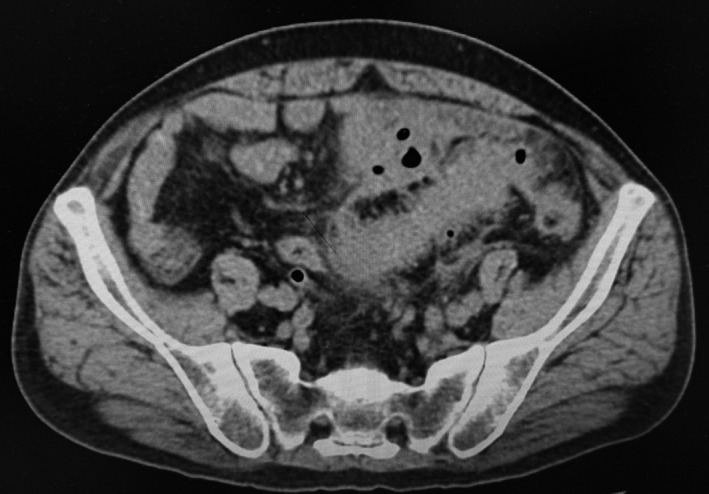
Computed tomography image shows pericolic abscess walled off by colon, mesocolon, bladder and pelvic peritoneum. This is Hinchey stage II. This patient was treated with antibiotics but failed. Sigmoid colon resection was carried out

About 20% of patients with diverticulitis develop a localized abscess at a pericolonic site (stage Ib) or in the pelvis or retroperitoneal space (stage II) (Figure 3). Most of these patients should be hospitalized and treated as inpatients. Conservative treatment with broad‐spectrum antibiotics is successful in up to 70% of those with small abscesses (<4 cm).^22^ If the abscess is larger or the conservative treatment fails, percutaneous drainage or surgery should be considered as a second‐line treatment. Percutaneous drainage is successful in up to 81% of patients.^21^ Percutaneous ultrasonography‐ or CT‐guided abscess drainage has been recognized as a standard therapeutic approach. Percutaneous drainage is often difficult, however, when there are small bowel loops adjacent to the fluid collection. Kosugi et al^23^ reported that a transluminal endoscopic approach through the perforated diverticulum is an easy, effective method for draining an abscess cavity. The drainage route is generally shorter than that from the transabdominal wall, and the risk of damage to the intestinal loop surrounding the abscess is reduced. At this stage of diverticulitis, surgery should be used as rescue therapy.^8^

### 4.3 Stages III‐IV

**Figure 4:**
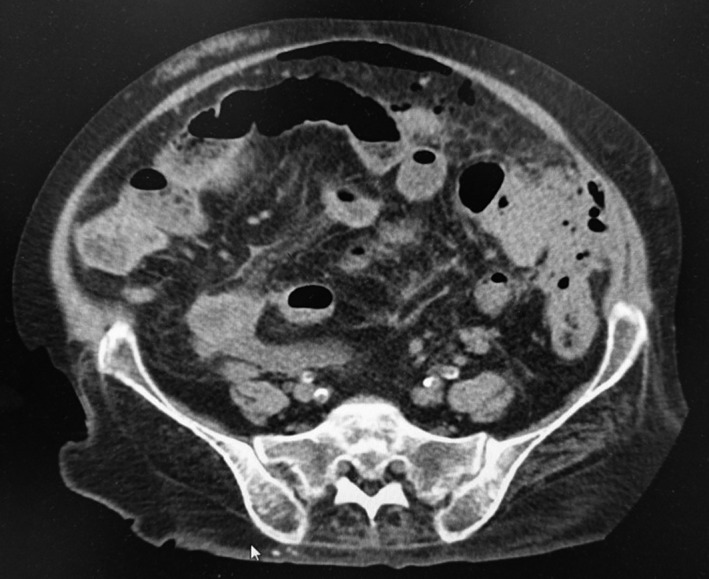
This is Hinchey stage III disease. Computed tomography image shows perforated diverticulitis of sigmoid colon with free air that caused generalized peritonitis. A two‐stage procedure, Hartmann's procedure was carried out

Approximately 6% of patients develop severe diverticulitis with perforation and generalized peritonitis (Figure 4).^18^ In the case of Hinchey stage III‐IV peritonitis, abdominal CT shows the presence of peritoneal fluid and extraluminal air. Clinically, symptoms of peritonitis (eg rebound tenderness, muscular defense) are found. Peritonitis is the most life‐threatening complication, with 14% mortality.^24^ Operative treatment is considered standard therapy for patients with Hinchey stage III or IV diverticulitis.

## 5 OPERATIVE THERAPY

Systematic surgical treatment of diverticulitis entails three‐stage surgery, as reported by the Mayo Clinic in 1907^25^: free drainage for perforated diverticulitis with addition of proximal colostomy; resection of the diseased segment; and closure of the ostomy. During the 1980s, Hartmann's procedure became the standard, and it is still widely practiced today. It is a two‐stage procedure involving resection of the diseased colon, closure of the distal rectal stump, and construction of an end‐colostomy. At the second stage, the colostomy is reversed. However, Hartmann's reversal, or stoma closure with reconstruction, may be difficult. It has been reported that the ostomy could not be closed in 30‐40% of cases owing to the high surgical risk in older patients with many comorbidities.^26^, ^27^

During the 1990s, one‐stage surgery was reported for patients in good general condition. It comprised primary resection with anastomosis, with or without protective ileostomy or colostomy.^28^ Recently, the safety and effectiveness of laparoscopic surgery for complicated diverticulitis has been reported. There was no mortality or serious complications directly related to the laparoscopic procedure, and laparoscopic colonic resection for Hinchey stage I‐II diverticulitis is at least as safe and effective as traditional open surgery.^29^ However, laparoscopic treatment of diverticulitis is technically challenging and requires adequate training and experience.^30^ The use of laparoscopic surgery for the emergency treatment of complicated diverticulitis is still controversial.^31^ The laparoscopic treatment of complicated diverticulitis has evolved and changed over the last decade. Increasing experience and availability of better equipment have made surgery faster and extended the capability of laparoscopic surgery for complicated disease.^32^ Results of the meta‐analysis indicate that laparoscopic surgery is associated with an overall lower mortality rate and a lower overall morbidity rate than open surgery for diverticulitis. However, laparoscopic surgery is associated with a higher rate of anastomotic bleeding compared to open surgery.^33^ There is no place today for laparoscopic resection for Hinchey stage III or IV disease.^9^

The usefulness of laparoscopic lavage without resection for complicated diverticulitis was reported, after which randomized, controlled trials were conducted. Laparoscopic lavage versus primary resection, however, did not reduce the incidence of severe postoperative complications and led to worse outcomes. For patients with Hinchey stage III disease, the superiority of laparoscopic lavage compared with colectomy is not proven, and rather serious complications were reported.^34^ The rate of reintervention within 30 days postoperatively was 28.3% in the lavage group and 8.8% in the resection group. Despite this risk, one of the major proposed advantages of laparoscopic lavage is avoidance of a stoma. Laparoscopic lavage leads to more reinterventions, but does not increase the 30‐ or 90‐day mortality rates compared with resection. The role of laparoscopic lavage in Hinchey III is yet unclear. Further studies are required to clarify its role.^35^

**Figure 5:**
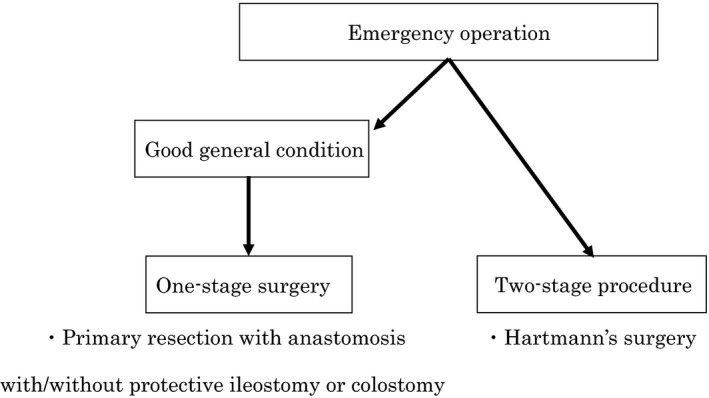
Operative treatment is considered standard therapy for severe diverticulitis with perforation and generalized peritonitis. The standard procedure is Hartmann's operation. In good general condition, one‐stage surgery can be considered. It comprises primary resection with anastomosis, with or without protective ileostomy or colostomy

Currently, the standard procedure is Hartmann's operation or primary anastomosis with or without a diverting ileostomy or colostomy (Figure 5).

## 6 RECURRENCE

When colonic diverticulitis is treated conservatively, the overall recurrence rate is reported to be 18.1%, and the mean interval to the onset of the recurrence is 4.7 ± 5.9 months.^18^ There is a tendency towards an escalating recurrence risk with an increasing number of recurrences. Recurrence risk more than doubled after one previous episode and increased gradually related to the number of recurrences.^36^

Recurrent episodes of diverticulitis mostly have a benign course. Only 5.5% of patients with hospitalizations for recurrent diverticulitis are subjected to emergency surgery.^37^ The recurrence of mild diverticulitis is not considered a risk factor for serious complications, such as perforation or abscess formation.^38^

According to the guidelines of the American Society of Colon and Rectal Surgeons,^39^ the number of repeated minor episodes of diverticulitis is not a factor determining the indication for surgery. For most patients who present with complicated diverticulitis, it is their first attack, and elective colon resection after conservative therapy might not decrease the need for later emergency surgery.^40^ Thus, elective surgery after recovery should be considered carefully and decisions made on a case‐by‐case basis.^39^ The DIRECT trial randomizes patients presenting with either recurrent (three or more recurrences within 2 years) or persistent abdominal complaints after an episode of left‐sided diverticulitis to either elective resection or conservative treatment. The authors of this trial concluded that elective sigmoidectomy leads to improved quality of life compared with conservative management. This study has several limitations (a possible placebo effect of surgery, difficulties in patient recruitment and non‐surgical measures for prevention of recurrent) and long‐term data are not yet available.^41^ The best treatment for recurrent diverticulitis is undetermined.

## 7 CONCLUSIONS

The present review summarizes the diagnosis and treatment of patients with acute diverticulitis. Figure 1 summarizes the current diagnostic and therapeutic options for colonic diverticulitis. It is thought that the prevalence of diverticulitis of the colon will increase in Japan in the future, as has occurred in Western countries. Because medical treatment of this entity will also increase, surgeons should be aware of a systematic strategy for treating the various stages of colonic diverticulitis appropriately.

## 8 DISCLOSURE

Conflict of Interest: Authors declare no conflicts of interest for this article.
